# Vascular Endothelial Growth Factor Receptor-3 Directly Interacts with Phosphatidylinositol 3-Kinase to Regulate Lymphangiogenesis

**DOI:** 10.1371/journal.pone.0039558

**Published:** 2012-06-22

**Authors:** Sanja Coso, Yiping Zeng, Kenneth Opeskin, Elizabeth D. Williams

**Affiliations:** 1 Centre for Cancer Research, Monash Institute of Medical Research, Monash University, Melbourne, Victoria, Australia; 2 Department of Pathology, University of Melbourne, Parkville, Victoria, Australia; 3 Department of Anatomical Pathology, St Vincent’s Hospital, Fitzroy, Victoria, Australia; University of Nebraska Medical Center, United States of America

## Abstract

**Background:**

Dysfunctional lymphatic vessel formation has been implicated in a number of pathological conditions including cancer metastasis, lymphedema, and impaired wound healing. The vascular endothelial growth factor (VEGF) family is a major regulator of lymphatic endothelial cell (LEC) function and lymphangiogenesis. Indeed, dissemination of malignant cells into the regional lymph nodes, a common occurrence in many cancers, is stimulated by VEGF family members. This effect is generally considered to be mediated via VEGFR-2 and VEGFR-3. However, the role of specific receptors and their downstream signaling pathways is not well understood.

**Methods and Results:**

Here we delineate the VEGF-C/VEGF receptor (VEGFR)-3 signaling pathway in LECs and show that VEGF-C induces activation of PI3K/Akt and MEK/Erk. Furthermore, activation of PI3K/Akt by VEGF-C/VEGFR-3 resulted in phosphorylation of P70S6K, eNOS, PLCγ1, and Erk1/2. Importantly, a direct interaction between PI3K and VEGFR-3 in LECs was demonstrated both *in vitro* and in clinical cancer specimens. This interaction was strongly associated with the presence of lymph node metastases in primary small cell carcinoma of the lung in clinical specimens. Blocking PI3K activity abolished VEGF-C-stimulated LEC tube formation and migration.

**Conclusions:**

Our findings demonstrate that specific VEGFR-3 signaling pathways are activated in LECs by VEGF-C. The importance of PI3K in VEGF-C/VEGFR-3-mediated lymphangiogenesis provides a potential therapeutic target for the inhibition of lymphatic metastasis.

## Introduction

Lymph node status is an important factor used in determining the stage of disease progression, a powerful predictor of patient survival, and informs treatment decisions. Whilst lymph node metastases are not directly responsible for cancer-related death, they are indicators that tumor has developed a metastatic phenotype. In addition, cancer cells may spread from the lymph nodes to distant organs, where they can develop a secondary tumor and perturb critical functions of that organ. Consistent with this, improved patient survival is observed upon removal of involved regional lymph nodes for a number of cancers [1]–[3]. Standard of care for solid tumors is the biopsy of the sentinel node (first lymph node which receives lymphatic drainage from the primary tumor) and, if indicated, extensive lymphadenectomy.

Entry of cancer cells into the lymphatic vasculature at the primary tumor site may be facilitated by the higher permeability of lymphatic vessels, and by the absence of a regular basement membrane [4]. Until recently, the presence of lymphatic vessels inside the tumor bulk was disputed [5], [6] with studies showing that peritumoral lymphatics are predominantly responsible for promoting metastasis [7], [8]. Furthermore, tumors can actively induce the formation of lymphatic vessels - typically via release of vascular endothelial growth factor (VEGF)-C or VEGF-D - and thereby promote metastasis to draining lymph nodes [9], [10].

Microvessel density, which includes both blood and lymphatic vessels, is an indicator of biological aggressiveness and metastatic potential in many types of solid tumors [11]. Intratumoral lymphatic vessels and metastasis to lymph nodes and lungs have been documented in mice bearing human tumor xenografts expressing VEGF-C or VEGF-D [9], [10], as well as in VEGF-C or -D transgenic mouse tumors [12].

The exact mechanism by which VEGF receptor (VEGFR) ligands increase tumor cell entry into lymphatic vessels is likely to involve several biological processes. The ligands might increase the surface area of functional lymphatics in the tumor margin, thus providing more opportunity for a tumor cell to enter the lymphatics and disseminate. Furthermore, VEGFR ligands may stimulate tumor-associated lymphatics or the draining lymph nodes to release chemotactic factors that recruit tumor cells to enter lymphatics, or they may directly affect tumor cells. Lymphatic endothelial cells (LECs) are ideally positioned to play a central role in the early steps of lymphangiogenesis as they express VEGFRs and respond to ligand stimulation *in vitro* [13] and *in vivo* [14].

VEGF-C is a primary regulator of lymphangiogenesis in developmental and pathological conditions including tumor lymphatic metastasis [15], [16]. Moreover, the expression of VEGF-C by tumor cells correlates with lymph node metastasis in a variety of human cancers, indicating an important role of this pathway in tumor progression (reviewed in [17]). VEGF-C binds and activates two related receptor tyrosine kinases, VEGFR-2 and VEGFR-3 [18], to induce lymphangiogenesis [19]–[21]. Despite the importance of the VEGF-C/VEGFR-3 pathway in lymphangiogenesis, its downstream signal transduction pathways have remained relatively unexplored. Thus far studies in lymphatic cancer metastasis have mostly investigated at VEGF-C/VEGFR-3 signaling pathways in the context of cancer cell signaling, such as breast cancer cells [22] and squamous cells carcinoma of the head and neck [23]. Because lymphatic vessels are an important indicator of disease in metastatic cancers and an essential treatment target, it is imperative to further explore the downstream signaling pathways regulating primary LECs and in lymphatic vessels associated with metastatic disease.

To delineate the signal transduction pathways involved in VEGFR-3-mediated lymphangiogenesis, we investigated the biological functions and signaling pathways stimulated by VEGF-C in primary LECs. The results show that VEGF-C stimulation induces distinct intracellular signaling pathway activation and biological responses in LECs. Only one study done by Makinen et al. [20] has indicated the importance of PI3K signaling in the growth and survival of VEGF stimulated LEC. However, it has not been reported thus far whether there is a direct interaction between PI3K and VEGFR-3 molecules in LECs and more importantly, there have been no studies to show a direct interaction of VEGFR-3/PI3K in isolated LECs nor lymphatic vessels *in situ*. Importantly, we show here for the first time that PI3K forms a complex with VEGFR-3 in isolated LECs, and that this complex is readily detected in lymphatic vessels (in addition to cancer cells) in lymph node metastatic small cell lung carcinoma (SCLC) specimens. Thus, this signaling complex may be a novel prognostic factor and suggests a critical role for PI3K in VEGF-C/VEGFR-3 induced lymphangiogenesis.

## Results

### VEGF-C Induces PI3K-dependent Akt Activation via VEGFR-3 in LECs

**Figure 1:**
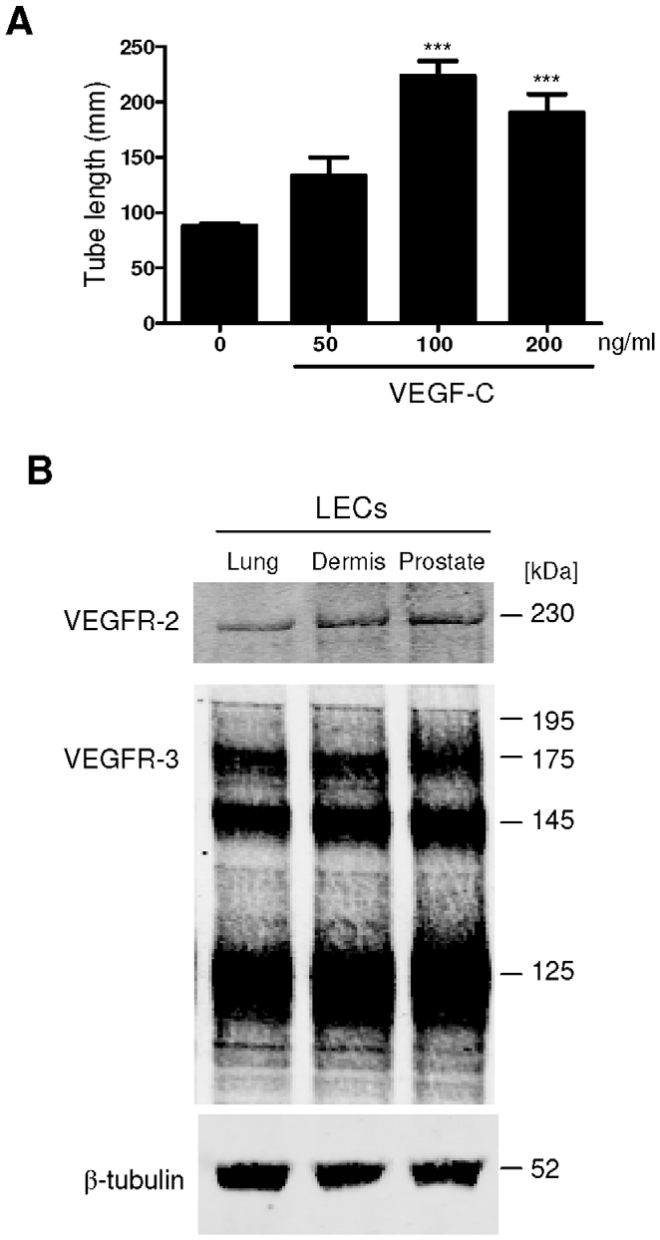
VEGF-C induces LEC tube formation. *A*, Prostatic LEC tube length at 4.5 hours post VEGF-C treatment was quantified using ImageJ. VEGF-C significantly increased the number of tubes formed compared to vehicle control. Data expressed as mean±s.e.m., n = 3, ***P<0.001 using One-way ANOVA, Bonferroni post-analysis. *B*, Western blotting analysis of VEGFR-2 and VEGFR-3 expression in lung, neonatal dermis and prostate LECs. β-tubulin was used as a loading control.

**Figure 2:**
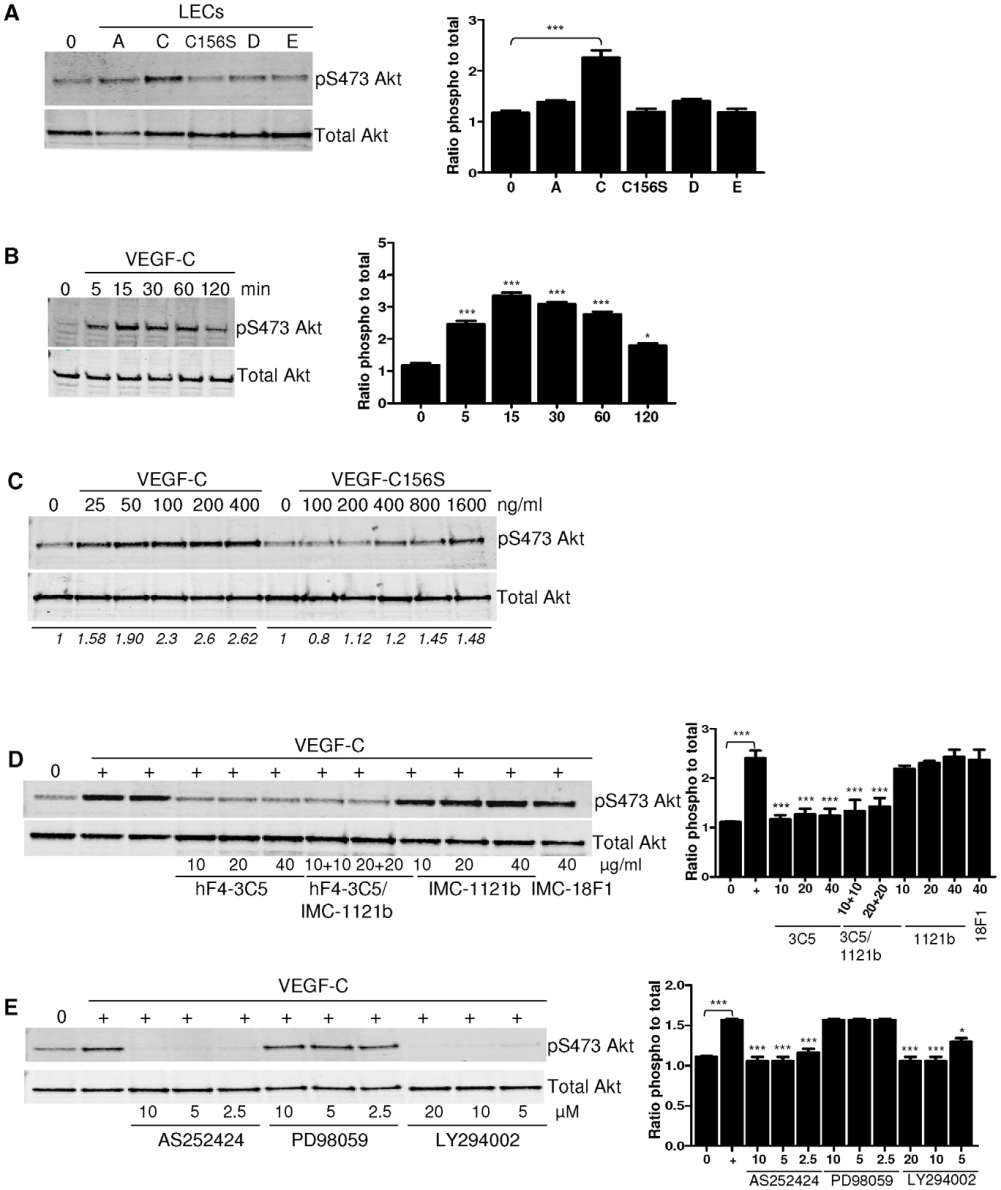
VEGF-C induces PI3K-dependent Akt phosphorylation via VEGFR-3 in LECs . *A*, Western blotting analysis of phosphorylated Akt (S473) in prostatic LECs following a 15 minute ligand stimulation: VEGF-A (100 ng/ml), VEGF-C (100 ng/ml), VEGF-C156S (250 ng/ml), VEGF-D (250 ng/ml) and VEGF-E (100 ng/ml). *B*, Time course for Akt phosphorylation (S473) in LECs after VEGF-C (100 ng/ml) stimulation. *C*, Concentration-dependent phosphorylation of LEC Akt (S473) following 15 minute stimulation with VEGF-C or VEGF-C156S. *D*, Effect of inhibition of VEGFR-3 (hF4-3C5), VEGFR-2 (IMC-1121b) or VEGFR-1 (IMC-18F1) on LEC Akt (S473) phosphorylation in response to VEGF-C (100 ng/ml). *E*, Effect of inhibition of PI3K (AS252424, LY294002) or Raf/MEK (PD98059) on Akt (S473) phosphorylation in LECs in response to VEGF-C (100 ng/ml). Serum-free vehicle treated LEC lysate is indicated by ‘0′ in all blots. n = 3. Densitometry analysis is shown for each blot either italicized or graphed; where integrated intensity of phosphorylated molecules was firstly compared to that of the total target protein for each sample, and then expressed as fold increase in integrated density compared to VEGF-C treated control samples. *P* value calculated using one-way ANOVA. For panels A-B data is compared to time-point zero; panels D-E data is compared to VEGF-C treated control, except where indicated for serum free/VEGF-C treatment comparison. Columns: mean; bars: s.e.m.; P<0.05 (*), P<0.01 (**); P<0.001 (***).

VEGF-C (at 100 and 200 ng/ml) significantly stimulated tube formation in lung, dermal and prostatic LECs (Figure 1A). VEGFR-2 and VEGFR-3 were abundantly expressed in LECs isolated from all tissues examined (lung, dermis, prostate; Figure 1B). As the PI3K/Akt signaling pathway has been reported to be downstream of both VEGFR-2 and VEGFR-3 [24], [25], we investigated the effect of different VEGFR ligands on Akt activation in a panel of prostatic LECs. VEGF-C, but not other VEGFR ligands, rapidly stimulated Akt phosphorylation at Ser473 (Figure 2A) in a time- and concentration-dependent manner (Figure 2B & C). VEGF-C-induced Akt phosphorylation was readily detectable following 5 minutes of treatment, with maximal phosphorylation reached after 15 minutes. Akt phosphorylation remained above control levels 120 minutes post VEGF-C (100 ng/ml) treatment (Figure 2B). VEGF-C156S, the VEGF-C mutant form which only activates VEGFR-3, also induced phosphorylation of Akt at high concentrations (Figure 2C). By contrast VEGF-A, which can activate both VEGFR-1 and VEGFR-2 (see Figure S1 for assessment of specificity), and VEGF-E, a VEGFR-2 specific ligand, did not activate Akt in LECs (Figure 2A).

As both VEGFR-2 and VEGFR-3 are receptors for VEGF-C, we sought to further define which receptor was involved in VEGF-C-induced Akt phosphorylation in LECs. Blocking VEGFR-3 using neutralizing antibody hF4-3C5 reduced VEGF-C-induced Akt activation to baseline levels. Neutralizing antibodies against either VEGFR-1 or VEGFR-2 had no effect on phospho-Akt levels (Figure 2D). Simultaneous inhibition of both VEGFR-3 and VEGFR-2 did not further increase the inhibition compared to blocking VEGFR-3 alone (Figure 2D). Thus VEGF-C activates Akt via VEGFR-3 in LECs. As Akt is a well documented downstream target of PI3K [26], we examined whether VEGF-C/VEGFR-3-induced Akt activation was PI3K dependent. The PI3K inhibitors LY294002 and AS252424, but not MEK1 inhibitor PD98059, abolished VEGF-C-induced Akt phosphorylation (Figure 2E), demonstrating that VEGF-C/VEGFR-3 mediates Akt phosphorylation via PI3K.

### P70S6K, eNOS and PLCγ, but not mTOR, are Activated by VEGF-C Signaling through VEGFR-3

**Figure 3:**
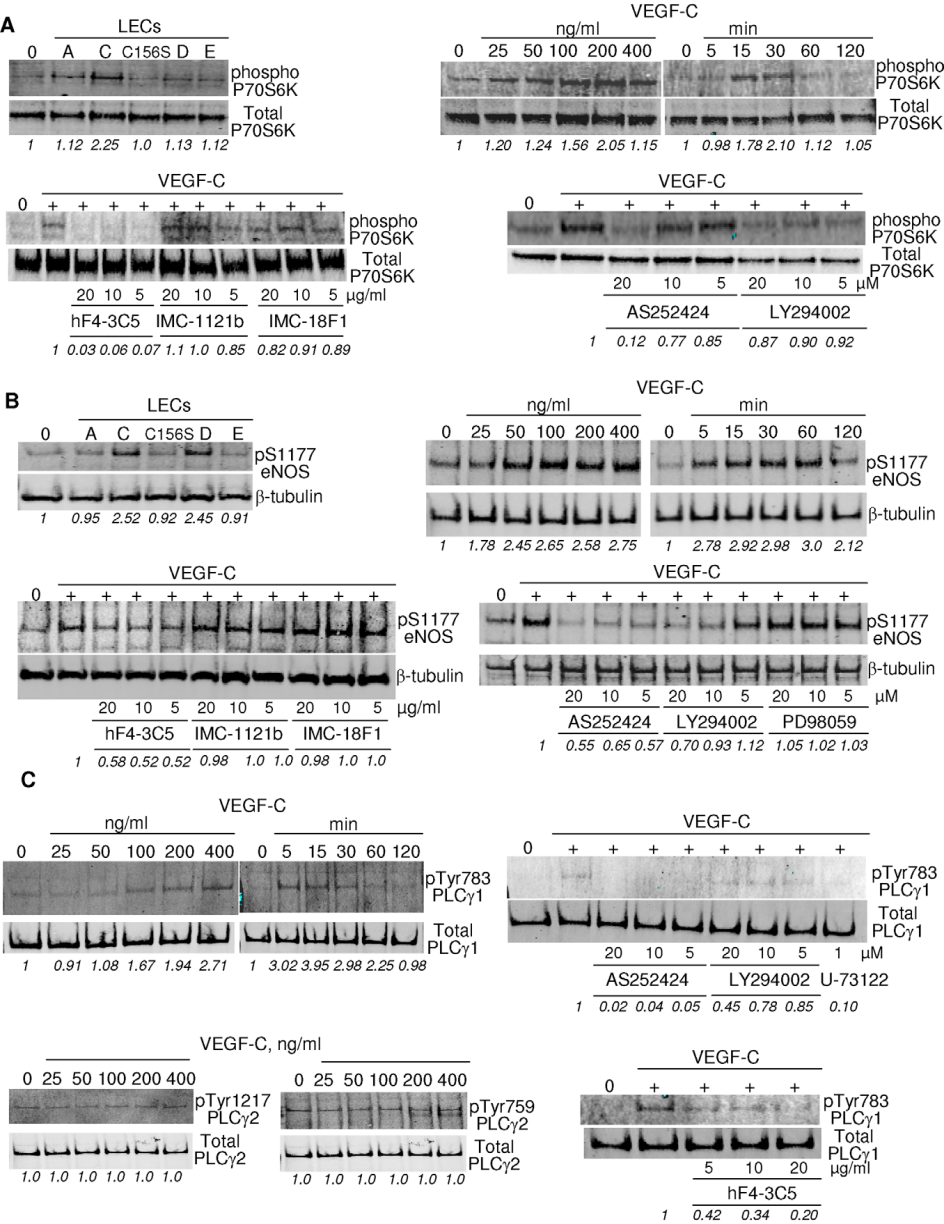
VEGF-C induces PI3Kγ-dependent P70S6K (A), eNOS (B), and PLCγ1 (C) phosphorylation via VEGFR-3 in LECs. Western blotting analysis of phosphorylated P70S6K (*A, top left*) and eNOS (S1177) (*B, top left*) in prostatic LECs following 15 minute stimulation with ligand: VEGF-C (100 ng/ml), VEGF-A (100 ng/ml), VEGF-C156S (250 ng/ml), VEGF-D (250 ng/ml) and VEGF-E (100 ng/ml). Time- and concentration-dependent phosphorylation of P70S6K (*A, top right*), eNOS (S1177) (*B, top right*), and PLCγ1 (Tyr783) (*C, top left*) in LECs in response to VEGF-C. The effect of inhibition of VEGFR-3 (hF4-3C5), VEGFR-2 (IMC-1121b) or VEGFR-1 (IMC-18F1) on phosphorylation of P70S6K (*A, bottom left*), eNOS (S1177) (*B, bottom left*); and VEGFR-3 (hF4-3C5) on PLCγ1 (Tyr783) (*C, bottom right*) on LEC response to VEGF-C (100 ng/ml). The effect of AS252424, LY294002, PD98059, and U-73122 on phosphorylation of P70S6K (*A, top right*), eNOS (S1177) (*B, bottom right*), and PLCγ1 (Tyr783) (*C, bottom right*) on LECs in response to VEGF-C. The effect of VEGF-C on phosphorylation of PLCγ2 (Tyr759, Tyr1217) in LECs (*C, bottom left*). Control serum-free vehicle treated LEC lysate is indicated by ‘0′ in all blots. n = 3. Densitometry analysis is shown under each blot in italics; where integrated intensity of phosphorylated molecules was firstly compared to that of the total target protein for each sample, and then expressed as fold change in integrated density compared to either serum-free (A, *top left and right panels*; B, *top left and right panels*; C, *top and bottom left panels*) or VEGF-C treated control samples (A, *bottom left and right panels*; B, *bottom left and right panels*; C, *bottom and bottom left panels*).

To identify the pathways downstream of Akt activation in response to VEGF-C, we examined the effects of VEGFR ligands on the activation of P70S6K and mammalian target of rapamycin (mTOR) in LECs. Phosphorylation of P70S6K was detected in LECs stimulated by VEGF-C (100 ng/ml), but not other members of VEGFR family (Figure 3A, top left). VEGF-C induced P70S6K phosphorylation in a concentration- and time-dependent manner, with maximal phosphorylation reached after 30 minute treatment (Figure 3A, top right). This stimulation pattern is similar to that of VEGF-C-induced Akt phosphorylation in LECs (Figure 2B & C), although P79S6K phosphorylation levels returned to baseline more quickly. Inhibition of VEGFR-3, but not VEGFR-1 or VEGFR-2, abolished VEGF-C-induced P70S6K phosphorylation (Figure 3A, bottom left). The highly selective PI3Kγ inhibitor AS252424, whose half-maximal inhibitory concentration (IC50) for isoform γ is over 200 times lower than that of LY294002 [27], decreased P70S6K phosphorylation in response to VEGF-C. By contrast, non-selective PI3K inhibitor LY294002 did not block VEGF-C-induced phosphorylation of P70S6K (Figure 3A, bottom right). These results suggest that PI3Kγ, rather than the other PI3K isoforms, is involved in VEGF-C-stimulated P70S6K phosphorylation. By contrast, none of the VEGFR ligands investigated altered the phosphorylation status of mTOR in LECs (Figure S2A). Together, these results show that VEGF-C activates stimulation of the VEGFR-3/PI3K/Akt/P70S6K pathway in LECs.

Akt activation also induces endothelial nitric oxide synthase (eNOS) phosphorylation in blood vascular endothelial cells in response to VEGF-A and VEGF-C [28], [29]. We determined the effect of VEGF-C on eNOS activation in LECs. Both VEGF-C and VEGF-D strongly phosphorylated eNOS at Ser1177 (Figure 3B, top left). By contrast, VEGF-A, VEGF-E and VEGF-C156S had only very weak effects on the phosphorylation of eNOS in LECs (Figure 3B, top left). VEGF-C induced eNOS phosphorylation at Ser1177 in a concentration- and time-dependent manner, with a maximal phosphorylation reached after 15 minutes of treatment (Figure 3B, top right). Inhibition of VEGFR-3, but not VEGFR-1 or VEGFR-2, reduced VEGF-C-induced eNOS Ser1177 phosphorylation (Figure 3B, bottom left). The PI3K inhibitors LY294002 and AS252424, but not the Raf/MEK inhibitor PD98059, abolished VEGF-C-induced eNOS Ser1177 phosphorylation in a concentration-dependent manner (Figure 3B, bottom right). VEGF-C stimulation induced the phosphorylation of Erk1/2 in LECs (Figure S2B-G). PD98059 diminished, but did not completely block VEGF-C-induced Erk1/2 phosphorylation in a dose-dependent manner (Figure S2D) indicating that Raf/MEK regulation of VEGF-C induced Erk phosphorylation may be indirect. PD98059 had no effect on VEGF-C-induced Akt phosphorylation (Figure 2E), indicating the specificity of this inhibitor to MAPK in LECs. At least two upstream pathways have been implicated in Raf/MEK/Erk activation including protein kinase C (PKC) and Ras [30]. To determine whether PKC is involved in Erk activation in primary LECs, cells were pretreated with a PKC inhibitor GF109203X prior to VEGF-C stimulation. GF109203X blocked VEGF-C-induced Erk1/2 activation dose-dependently (Figure S2D), indicating PKC mediates VEGF-C/VEGFR-3-induced Raf/MEK/Erk activation in LECs. The specificity of the inhibitory effect of GF109203X on PKC in LECs was confirmed by a lack of effect on Akt activation in LECs (data not shown). To address whether PI3K is involved in VEGF-C-induced Erk activation, LECs were pretreated with the PI3K inhibitor AS252424 prior to VEGF-C stimulation (Figure S2D). Our data shows that VEGF-C/VEGFR-3 interaction stimulates eNOS phosphorylation in LECs via the PI3K/Akt pathway.

The PI3K and phospholipase Cγ (PLCγ) pathways are interconnected. PLCγ is known to be involved in a number of tyrosine kinase signaling pathways, including the VEGF-A/VEGFR-2 cascade [31]–[33]. To address whether PLCγ is involved in the VEGF-C signaling pathway and whether this is dependent on isoform PLCγ1 or PLCγ2 [34], LECs were treated with various concentrations of VEGF-C for 15 minutes and PLCγ phosphorylation assessed. As shown in Figure 3C (top left), VEGF-C stimulated PLCγ1 phosphorylation at tyrosine 783 in a concentration-dependent manner, whereas it had no effect on the phosphorylation of PLCγ2 at either tyrosine 1217 or tyrosine 759 (Figure 3C, bottom left). Maximal phosphorylation of PLCγ1 was reached after a 15 minute stimulation with VEGF-C, and declined rapidly (Figure 3C, top left). Blocking VEGFR-3 inhibited VEGF-C-induced PLCγ1 phosphorylation (Figure 3C, bottom right). These results show that VEGF-C phosphorylates PLCγ1 via VEGFR-3 in LECs. A direct physical association of VEGFR-3 with PLCγ1 was not detected using co-immunoprecipitation (data not shown). As PLCγ1 has been reported to be a PI3K downstream target [35], we investigated whether VEGF-C/VEGFR-3 induced LEC PLCγ1 phosphorylation is PI3K dependent by pretreating LECs with PI3K inhibitor prior to VEGF-C stimulation. The selective PI3Kγ inhibitor AS252424 decreased VEGF-C-induced PLCγ1 phosphorylation (Figure 3C, top right), whereas the non-selective PI3K inhibitor LY294002 had no effect. On the other hand, silencing PLCγ in LECs using siRNA had no effect on VEGF-C-induced Akt phosphorylation (Figure S3). Together, these results demonstrate the involvement of PI3Kγ in LEC PLCγ1 activation in response to VEGF-C stimulation, whereas VEGF-C-induced Akt phosphorylation is not dependent on PLCγ1.

### Blocking PI3K Inhibits VEGF-C-induced LEC Tube Formation and Migration

**Figure 4:**
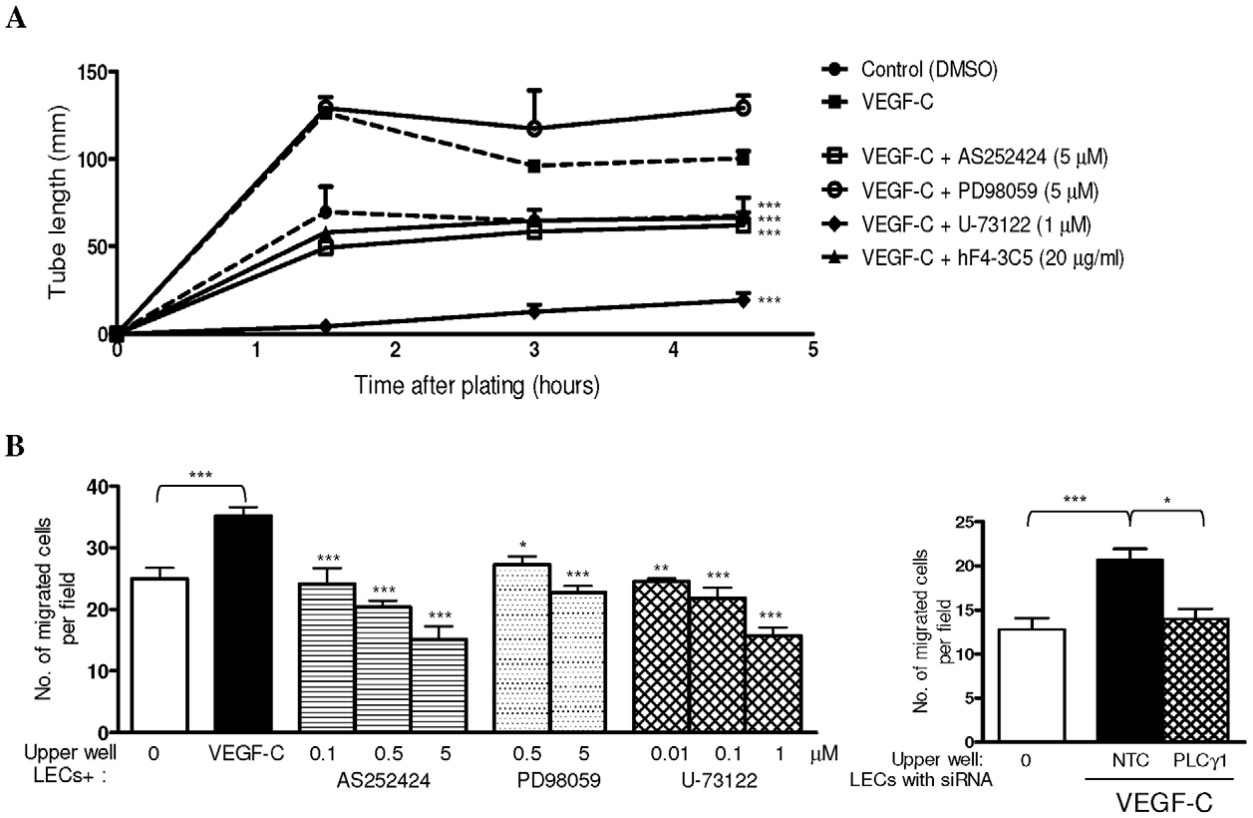
Blocking PI3K inhibits VEGF-C-induced LEC tube formation and migration. Effect of anti-VEGFR-3 antibody (hF4-3C5, 20 µg/ml), selective PI3Kγ inhibitor (AS252424, 5 µM), Raf/MEK inhibitor (PD98059, 5 µM) and PLCγ1 inhibitor (U-73122, 1 µM) on VEGF-C-induced (200 ng/ml) LEC tube formation (*A*) and migration (*B*, *left panel*). Effect of PLCγ1 siRNA or non-targeting control (NTC) on LEC VEGF-C-induced migration (*B*, *right panel*). Control serum-free (with DMSO as appropriate) LEC is indicated by ‘0′. *P<0.05, **P<0.01, ***P<0.001, compared to VEGF-C treated cells/NTC. n = 3. Data shown as mean±s.e.m.

To investigate the functional role of PI3K/Akt, PLCγ and Raf/MEK/Erk pathways in VEGF-C/VEGFR-3-induced lymphangiogenesis, we examined the effect of inhibiting these pathways on VEGF-C-induced LEC tube formation. As shown in Figure 4A, a neutralizing antibody against VEGFR-3 (hF4-3C5) reduced VEGF-C-induced capillary-like network formation in LECs to basal levels, demonstrating VEGF-C induces LEC differentiation and migration via VEGFR-3. Similarly, inhibition of PI3K/Akt activity suppressed VEGF-C-induced LEC tube formation to basal levels. PLCγ inhibition had a profound effect, almost completely inhibiting all tube formation. By contrast, the Raf/MEK inhibitor had no effect on LEC tube formation induced by VEGF-C (Figure 4A). These data show that VEGF-C/VEGFR-3-induced LEC capillary-like structure formation depends on PI3K/Akt and PI3K/PLCγ pathways, but not the Raf/MEK/Erk pathway.

We also determined the role of PLCγ, PI3K/Akt and Raf/MEK/Erk pathways in VEGF-C-induced LEC migration. As shown in Figure 4B, VEGF-C significantly stimulated LEC migration (*P*<0.001). Pharmacological inhibition of either PI3K/Akt, Raf/MEK/Erk or PLCγ blocked VEGF-C-induced LEC migration in a concentration-dependent manner (Figure 4B, left panel). Moreover, silencing PLCγ1 in LECs using siRNA significantly reduced VEGF-C-induced LEC migration (Figure 4B, right panel). These results indicate that PI3K/Akt, PI3K/PLCγ1, and Raf/MEK/Erk pathways are involved in VEGF-C-induced LEC migration.

### VEGFR-3 and PI3K Form a Complex

**Figure 5:**
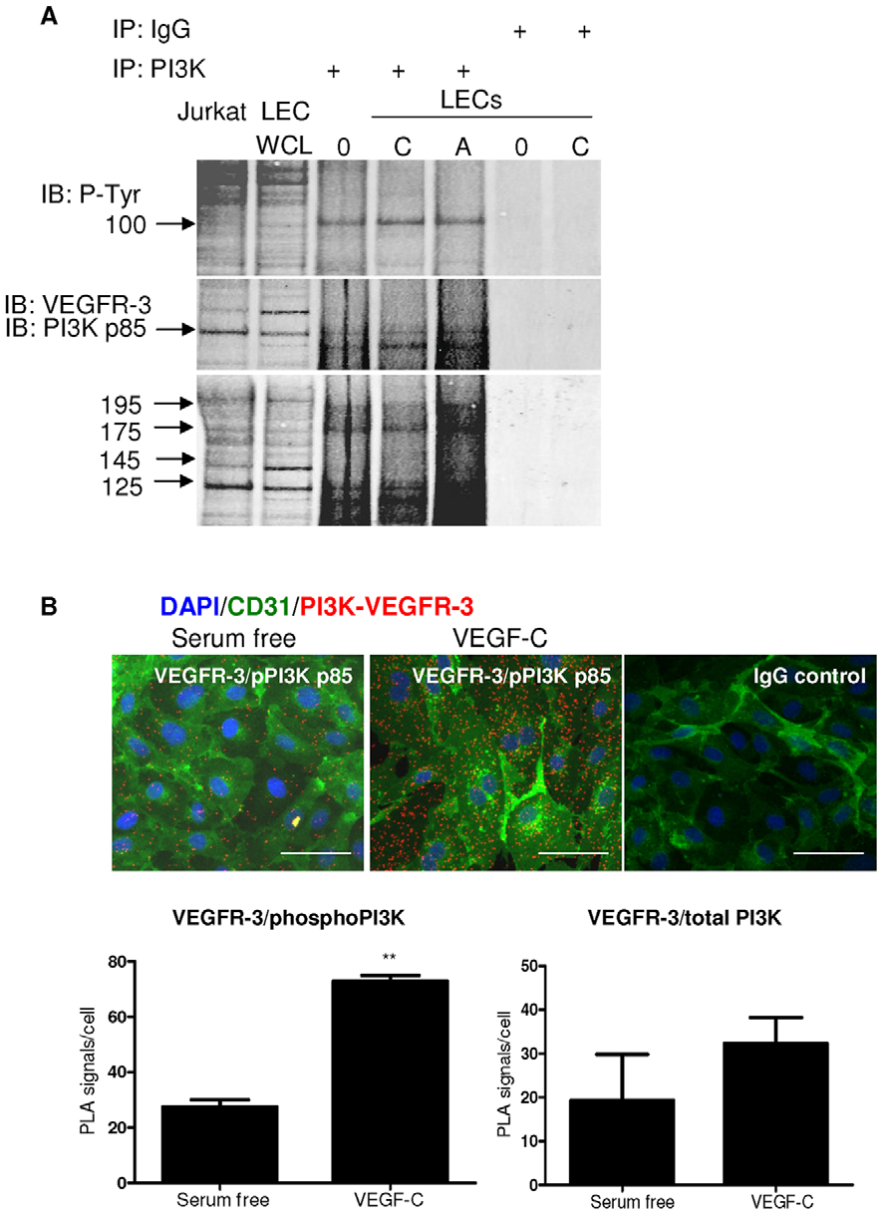
Direct interaction between PI3K p85 and VEGFR-3. *A,* LEC whole cell lysate was immunoprecipitated with anti-PI3K or isotype control IgG followed by Western blotting of phospho-tyrosine (p-Tyr), VEGFR-3 or PI3K. LECs were treated with vehicle control (serum-free media ‘0′), IgG control, VEGF-C (100 ng/ml, C) or VEGF-A (100 ng/ml, A) ligand for 15 minutes. Control untreated LEC whole cell lysate is indicate by ‘WCL’ and Jurkat cell lysate served as a positive control (Jurkat). *B,* Detection of VEGFR-3/phospho-PI3K complexes (*red spots*) *in vitro* in LECs grown in absence (*B*, *upper left panel*) or presence (*B, upper middle panel*) of VEGF-C (100 ng/ml) for 15 minutes; CD31 staining (*green*); DAPI (*blue*); isotype IgG control in PLA (*B, upper right panel*); quantification of VEGFR-3/phospho-PI3K and VEGFR-3/total-PI3K PLA signals using Olink Imaging Software (*B*, *lower panel*). Data shown as mean±s.e.m. **P<0.01 using t-test analysis. n = 3. Bar = 50 µm.

Due to the critical role of PI3K in all VEGFR-3-mediated pathways, we determined the interactions between VEGFR-3 and PI3K using a co-immunoprecipitation strategy. As shown in Figure 5A, there was direct association between total VEGFR-3 and PI3K subunit in LECs irrespective of ligand stimulation, indicating that this association was constitutive under tissue culture conditions.

To further extend this finding, we employed *in situ* proximity ligation assay (PLA), which is highly specific and sensitive in depicting close proximity of cellular molecules [36]. A significant increase in VEGFR-3/phosphoPI3K interactions was detected in LECs treated with VEGF-C when compared to vehicle control (Figure 5B). No interaction signals were detected in negative controls, which included the use of isotype control IgGs (Figure 5B) and omission of one or both of the primary antibodies (data not shown).

### VEGFR-3/PI3K Complexes Distinguish Lymph Node Metastatic Small Cell Lung Cancers from Non-metastatic Tumors

**Figure 6:**
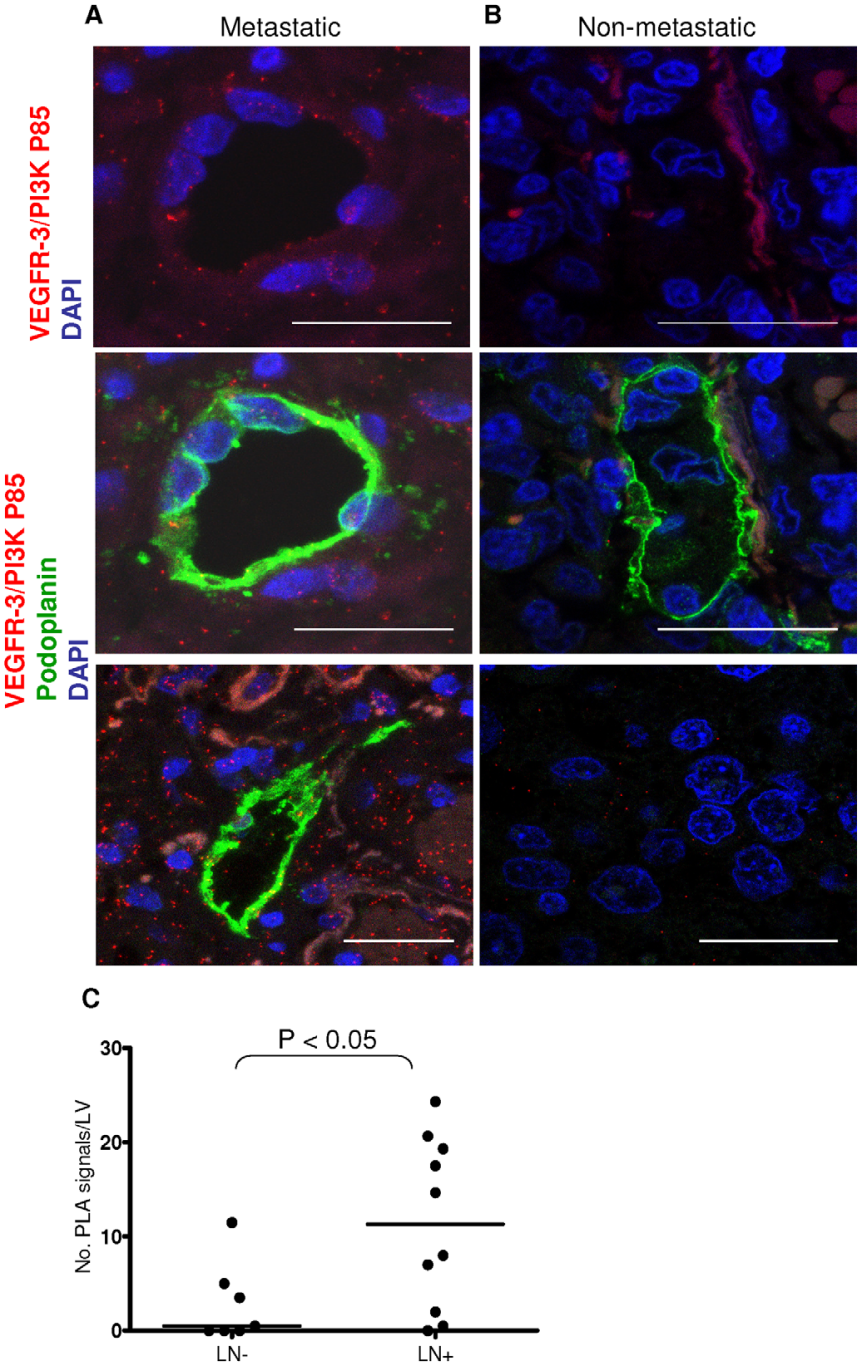
Direct interaction between PI3K p85 and VEGFR-3 in metastatic small cell lung carcinoma. VEGFR-3/PI3K complexes (*red*) detected by *in situ* PLA in lung tumor area and lymphatic vessels (*green,* podoplanin). Lymphatic vessel VEGFR-3/PI3K signals (*red*) and their representative co-staining with podoplanin (*green*) in metastatic (*A, top and middle panel,* respectively) and non-metastatic (*B, top and middle panel,* respectively) in small cell lung cancer tissue. VEGFR-3/PI3K signals were detected in cancer cells surrounding the lymphatic vessels in the metastatic samples (*A*, *top and bottom panel*) as well as the lymphatic vessels; whereas the low signal in non-metastatic samples was detected mostly away from the lymphatic vessels (*B*, *bottom panel*), and not in tumor cells inside or surrounding the non-metastatic lymphatic vessels (*B*, *top and middle panels*). Bar, 25 µm. DAPI (*blue*); *C.* Quantification of PLA signals (at least 5 different regions in each sample) in the lymphatic vessels (LV) (*C*, *left*) in lymph node negative (LN-; n = 7; two samples were excluded because there were no lymphatic vessels present in the sections examined) and lymph node positive (LN+; n = 10) samples using Olink Imaging Software. Plotted are mean values for individual patients. Line represents median value. *P<0.05.

**Figure 7:**
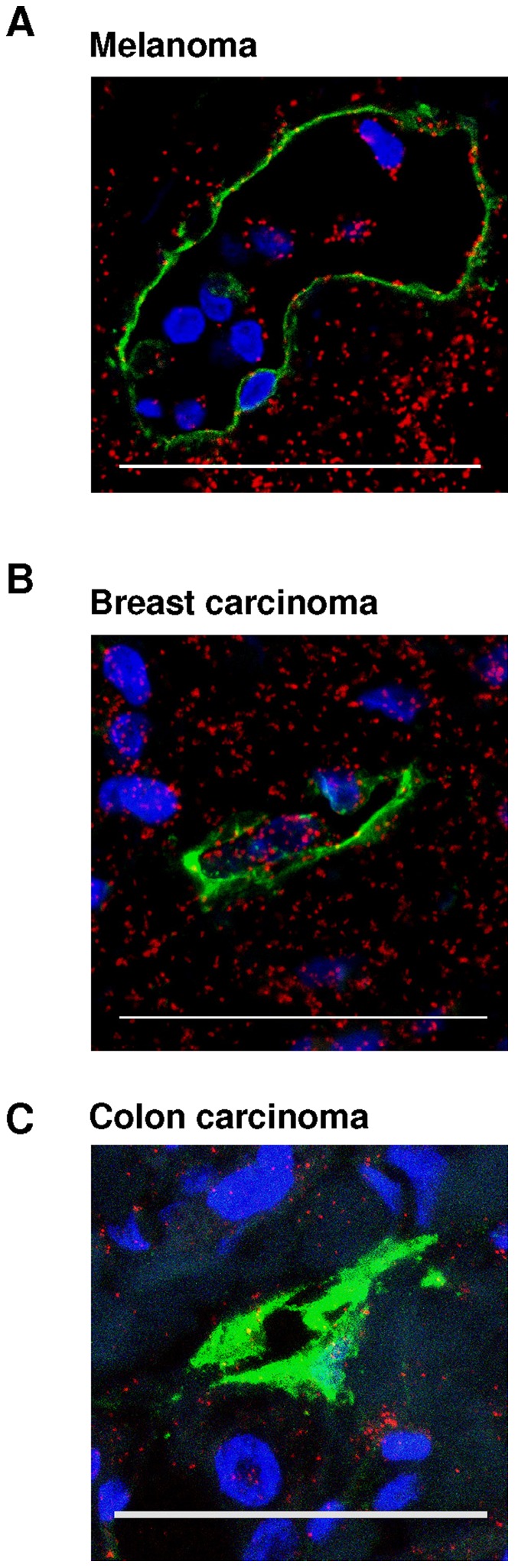
Direct interaction between PI3K p85 and VEGFR-3 in metastatic melanoma (A), breast (B) and colon (C) cancers. PI3K p85/VEGFR-3 complexes (*red*) detected by *in situ* PLA in tumor cells and lymphatic vessels (*green,* podoplanin). Bar, 50 µm. Nuclei stained with DAPI (*blue*).

We next used *in situ* PLA to investigate the interactions between VEGFR-3 and PI3K in human tumors. PLA VEGFR-3/PI3K signals were significantly higher in lymphatic vessels of primary small cell lung carcinomas from patients with lymph node metastatic tumors (Figure 6A, C) when compared to patients with non-metastatic tumors (Figure 6B, C). PLA VEGFR-3/PI3K signals were also detected in cancer cells surrounding the lymphatic vessels in the metastatic samples (Figure 6A, *top and bottom panels*), whereas in non-metastatic samples they were detected mostly in cells distant to the lymphatic vessels (Fig, 6B, *right bottom panel*), but rarely in those containing within or surrounding the non-metastatic lymphatic vessels (Figure 6B, *top right panel*). The VEGFR-3/PI3K interaction was also observed in lymphatic vessels (and the tumor cells) of lymph node metastatic melanoma, breast and colon tissue (Figure 7). These results suggest that VEGFR-3/PI3K p85 complex formation indicates “tumor activated” lymphatic endothelium *in vivo*.

## Discussion

Activation of the PI3K pathway is a common observation in human tumors (reviewed in [37], [38]). We have shown here for the first time PI3K is directly associated with VEGFR-3 in LECs in both isolated cells and human tumors. In the current study, we used primary LECs to dissect the VEGF-C/VEGFR-3 signaling pathway. VEGF-C selectively promoted tube formation in LECs via activation of two classic signaling pathways, PI3K/Akt and PLCγ consistent with a previous study [20]. Interestingly, VEGF-E (VEGFR-2 specific ligand) stimulation induced the phosphorylation of Erk1/2 in LECs, but had no effect on the activation of Akt or its downstream targets P70S6K or eNOS, despite the expression of VEGFR-2 in LECs. Taken together, these results reflect distinct biological responses to growth factor stimulation that is specific the ligand. The molecular basis for this remains to be determined.

The serine/threonine protein kinase Akt is a critical molecule in the cellular signaling pathways leading to angiogenesis [39], [40]. However, little is known about its role in lymphangiogenesis. In this study we demonstrated in LECs that VEGF-C binding to its receptor VEGFR-3 induced activation of Akt via PI3K. By contrast, VEGF-A stimulation had a much lesser effect on Akt phosphorylation despite the expression of its cognate receptor, reported also in a recent study by Dellinger et al. [33]. The Akt pathway was also shown to be important for survival, growth and migration of human microvascular endothelial cells by Makinen et al. [20].

We also found that P70S6K and eNOS serve as downstream targets of the PI3K/Akt pathway in response to VEGF-C. VEGF-A induced a small increase in eNOS (in line with Lahdernanta et al. [41]), as well as P70S6K expression (which has not been shown before). Kobayashi et al. [42] have demonstrated VEGF-C mediated P70S6K/mTOR inhibitor rapamycin leads to reduction of lymphatic metastasis using lymphatic metastasis-prone murine pancreatic tumor cells. We show here for the first time the importance of PI3K/Akt/P70S6K/eNOS pathway in regulating lymphangiogenic properties in LECs.

In addition, we showed for the first time a physical interaction between VEGFR-3 and PI3K in LECs that was independent of ligand (VEGF-C) stimulation. In clinical specimens VEGFR-3/PI3K interaction was associated with lymph node metastasis suggesting that this complex may play a critical role in tumor-induced lymphangiogenesis and thus the subsequent development of lymph node metastasis.

Blocking PI3K abolished VEGF-C-stimulated LEC tube formation and migration, demonstrating that the PI3K/Akt signaling pathway plays a central role in LEC differentiation and migration induced by VEGF-C/VEGFR-3, possibly via substrates such as P70S6K and/or eNOS. Consistent with this, a central role for the PI3K/Akt pathway has also been demonstrated in LEC tube formation stimulated by the non-VEGF family lymphangiogenic factors fibroblast growth factor-2 (FGF-2) and endothelin-1 [43]. Furthermore, eNOS has recently been reported to serve as a mediator of VEGF-C-induced lymphangiogenesis [44]. In rat LECs, FGF-2 stimulated tube formation is mediated by the PI3K/mTOR pathway [45].

Taken together, the signaling pathway results show that activation of discrete intracellular signaling pathways results in specific biological responses in LECs. The exact molecular basis underpinning how each ligand generates a specific biological response in LECs remains to be determined. In addition, we demonstrate that PI3K/Akt/P70S6K, PI3K/Akt/eNOS, PI3K/PLCγ1 and PI3K/Raf/MEK/Erk are important downstream pathways for VEGF-C/VEGFR-3 signaling as each plays a role in LEC migration.

Our finding of elevated numbers of VEGFR-3/PI3K complexes in lymph node metastatic SCLC compared to organ confined samples, raises the possibility that measurement of signaling complexes such as VEGFR-3/PI3K interaction may be useful prognostic markers. There is currently limited data on the significance of the VEGF family in lymphatic vessels in metastatic SCLC, and our study provides the first evidence of the existence of VEGFR-3/PI3K in both tumor cells and lymphatic vessels. Tanno et al. [46] showed that SCLC tumor cells express functional VEGFR-2 and VEGFR-3. In non-small cell lung carcinoma (NSCLC) increased levels of VEGF-C and VEGF-D have been shown to correlate with lymphatic invasion and lymph node metastasis in patient samples [47], [48]. Arinaga et al. [49] have shown that NSCLC patients with tumors positive for VEGF-C and VEGFR-3 expression had decreased survival rates. An interesting study from Renyi-Vamos et al. [50] found that lymphangiogenesis in NSCLC negatively correlated with patient survival, but only in nonangiogenic tumors. These tumors mainly co-opt host tissue lymphatics during their growth, in contrast to the majority of angiogenic tumors in which expansion is associated with a concomitant lymphangiogenesis.

PI3K pathway inhibitors are just entering the clinic for cancer treatment of cancers following their success in pre-clinical murine models. The efficacy of PI3K inhibitors is generally attributed to a direct effect on cancer cells [51]. Our study indicates that targeting the PI3K pathway in the context of the lymphangiogenic pathway (VEGFR-3) may provide additional clinical benefit via the inhibition of lymph node metastasis. Further elucidation of the exact role of PI3K downstream targets in lymphangiogenesis *in vivo* will lead to a better understanding of the molecular mechanisms involved and will provide avenues for the development of potential therapeutic drugs and combinational approaches targeting pathological lymphangiogenesis.

## Materials and Methods

### Ethics Statement

These studies were conducted with ethical approval of the St. Vincent’s Hospital Human Research Ethics Committee HREC-A and Monash University Standing Committee on Ethics in Research Involving Humans, and in accordance with Australian National Health and Medical Research Council Guidelines. Written informed consent for the collection of fresh tissue and generation of cell lines was obtained. Informed consent was waived by the Ethics committee for the archival tissues used for immunohistochemical analyses as these were used anonymously.

### Reagents

Neutralizing antibodies specific to human VEGFR-1 (IMC-18F1), human VEGFR-2 (IMC-1121b), and human VEGFR-3 (hF4-3C5) were obtained from ImClone (NY). Antibodies against phosphorylated proteins P70S6K (Thr389, Clone: 1A5, Cat. no. 9206), mTOR (Ser2448, Cat. no. 2971), eNOS (Ser1177; Clone: C9C3, Cat. no. 9570), PLCγ1 (Tyr783; Cat. no. 2821), PLCγ2 (Tyr1217; Cat. no. 3871), PLCγ2 (Tyr759; Cat. no. 3874), Erk1/2 (Thr202/Tyr204; Cat no. 9101), Tyr-100 (Cat. no. 9411) and Akt (Ser 473; Cat. no. 9271S) were purchased from Cell Signaling Technology (MA). Antibodies against total P70S6K (Clone: 49D7, Cat. no. 9208), mTOR (Cat. no. 2972), eNOS (Clone: 49G3, Cat. no. 9586), PLCγ1 (Clone: D9H10, Cat. no. 5690), PLCγ2 (Cat. no. 3872), Erk1/2 (Clone: 137F5; Cat. no. 4695), and Akt (Cat. no. 9272) were purchased from Cell Signaling Technology (MA). The other antibodies used were: anti-phospho PI3K p85 (Tyr458/p55 Tyr199; Cat. no. 4228; Millipore, MA) and total PI3K p85 (Cat. no. 06–19; Millipore); mouse anti-human CD31 (Clone: JC70A, Cat. no. M0823; DAKO, Denmark); mouse anti-human podoplanin (Clone: D2-40, Cat. no. 730-23; Signet Laboratories, MA); mouse anti-human VEGFR-2 (Cat. no. AF357; R&D Systems, MN), goat anti-human VEGFR-3 (Cat. no. AF349; R&D Systems); mouse anti-human pan-actin (Clone: Ab-5, NeoMarkers, CA); Alexa Fluor 488- and 568-conjugated secondary antibodies (Invitrogen, CA), and IRDye700DX conjugated anti-mouse IgG and IRDye800CW conjugated anti-rabbit IgG (Rockland Immunochemicals, PA).

Recombinant human VEGF-A165 (Cat. no. 293-VE), VEGF-C (Cat. no. 2179-VC), VEGF-C Cys156Ser (Cat. no. 752-VC/CF), VEGF-D (Cat. no. 622-VD) and hepatocyte growth factor (HGF; Cat. no. 294-HG) were purchased from R&D Systems.

LY294002 and GF109203X were purchased from Calbiochem (CA). AS252424 and U-73122 were purchased from Alexis Biochemicals (NY). PD98059 was purchased from Cayman Chemical (MI).

Jurkat cell lysate was purchased from Millipore.

### Clinical Specimens

Archival formalin-fixed paraffin-embedded tissue blocks were retrieved from the St. Vincent’s Pathology archive for 19 patients diagnosed with small cell lung carcinoma (SCLC). Nine specimens were from patients with organ-confined disease, while the remaining ten specimens were from patients with lymph node metastases (median number of positive nodes 4.0, range 1–29). The patient characteristics are described in Table S1, and no statistically significant differences were observed between the two cohorts. Original diagnoses were confirmed on hematoxylin and eosin stained sections at the Department of Pathology, St. Vincent’s Hospital, Melbourne. Melanoma, colon and breast cancer specimens were also obtained from St. Vincent’s Hospital, Melbourne.

### Cell Culture

Primary LECs were isolated from human prostate tissue and characterized as previously described [13]. Pure (>99.9%) LEC populations from the sub-confluent primary cultures were achieved using the CD34 and CD31 combined selection method as previously described [13] (Figure S4). Primary neonatal human dermal LECs [52] and primary human lung LECs [53] were purchased from Lonza (Switzerland) and maintained in EGM™-2 MV medium (Lonza). LECs between passages 3 and 6 were used for the experiments described herein. Cells were routinely tested for mycoplasma using the MycoAlert Mycoplasma Detection kit (Lonza), and were always negative.

### LEC Treatment, Immunoprecipitation, and Western Blotting Analyses

LECs were plated in 12-well plates at a density of 10^5^ cells per well in EGM™-2 MV medium. After overnight attachment, cells were serum-starved (4 hours) and stimulated as indicated. The following growth factors were tested: VEGF-A (25–400 ng/ml), VEGF-C (25–400 ng/ml), VEGF-C156S (100–1600 ng/ml), VEGF-D (250 ng/ml) and VEGF-E (25–400 ng/ml). For blocking experiments, cells were pretreated for 1 hour with LY294002 (5–20 µM), AS252424 (2.5–20 µM), U-73122 (0.1–10 µM), PD98059 (2.5–10 µM), IMC-18F1 (5–20 µg/ml), IMC-1121b (5–20 µg/ml), or hF4-3C5 (5–20 µg/ml) prior to ligand stimulation. The neutralizing activity of IMC-1121b, and the lack of effect of hF4-3C5, on inhibition of total and phospho-VEGFR-2 were confirmed by western blotting analysis (Figure S1). Dimethylsulfoxide (DMSO), in which the inhibitors were dissolved, was used as vehicle control. After stimulation, cells were lysed in radioimmuno precipitation assay (RIPA buffer) (Cat. no. 89900, Thermo Scientific, CA) containing protease (Cat. no. 78410, Thermo Scientific) and phosphatase inhibitors (Cat. no. 78420, Thermo Scientific). For immunoprecipitation, equal amounts of whole cell lysate were incubated with anti-PI3K p85 or isotype IgG and protein pulled down using Protein A/G Plus-agarose beads (Santa Cruz, CA). Protein samples were separated by SDS-PAGE, transferred to nitrocellulose and immunoblotted with the antibodies indicated. Signals were visualized using an Odyssey Infrared Imaging System (LI-COR Biosciences, NE). Densitometry analyses of Western blotting results were performed using Odyssey software. Integrated intensity of phosphorylated molecules was firstly compared to that of the total target protein for each sample, and then expressed as fold increase in integrated density compared to VEGF-C treated control samples.

The experiments were performed using primary human lung, neonatal skin and prostate LECs, as well as Jurkat cells.

### Knockdown of PLCγ1 in LECs Using siRNA

The siRNA ON-TARGETplus SMARTpool PLCγ1 and the siRNA nonspecific control were obtained from Dharmacon (CO). LECs were plated in a 6-well plate and cultured for 24 hours to 50–60% confluence. Cells were transfected with 100 nM siRNA using Lipofectamine^TM^2000 (Invitrogen) as per the manufacturer’s protocol. VEGF-C stimulation and migration assays were performed 72 hours after siRNA transfection. The degree of knockdown was determined using parallel samples for protein analysis.

### Tube Formation Assay

LEC tube formation assays were performed using growth factor reduced (GFR) Matrigel™ (BD Biosciences) as previously described [13]. Growth factors, inhibitors or neutralizing antibodies were added at the start of the experiment. The reagents used were VEGF-A (50–200 ng/ml), VEGF-C (50–200 ng/ml), and anti-VEGFR-3 antibody (20 µg/ml). Images from all tube formation experiments were digitally captured using an inverted microscope (Olympus, Japan) at 1.5, 3, and 4.5 hours after plating. The total length of tube-like structures formed by LECs in each well was measured using Image J (NIH). Each treatment was performed in duplicate and the experiment repeated 3 times using different batches of LECs (dermis, lung and prostate).

### Cell Migration Assay

Migration assays were performed using 48-well microchemotaxis chambers (Neuroprobe Inc, MD). Polycarbonate filters (8 µm pore size; Neuroprobe) were coated with 100 µg/ml collagen type I (Sigma-Aldrich, MO) overnight at 4°C and air dried. The lower chamber wells contained serum-free EGM™-2 MV medium/0.1% BSA in the presence or absence of VEGF-C, and were covered by the filter. For inhibitor blocking experiments, monolayer LECs were serum starved (4 hours), cells were then lifted and resuspended at 10^4^ cells per 56 µl of EGM™-2 MV containing inhibitor or concentration-matched DMSO. After 30-minute pretreatment, cells were washed in phosphate buffered saline (PBS) and added to the upper chamber at 10^4^ cells per well. The cells were allowed to migrate for 6 hours at 37°C, after which time the filter was fixed with cold methanol and stained with Quick Dip (Fronine, Australia). Non-migrated cells on the upper surface of the filter were removed, and the number of migrated cells was digitally recorded (Leica) and 5 random fields counted using Image J (NIH). The assays were run in triplicate and repeated with four different batches of primary prostate LECs.

### Proximity Ligation Assay

Cells were plated into 8-well chamber slides (Lab-Tek II, Nalge Nunc International, Australia) at a density of 2×10^4^ cells/well. The following day, LECs were serum starved (4 hours) prior to treatment with VEGF-C (100 ng/ml) for 15 minutes. LECs were then fixed in 10% formalin and subjected to *in situ* PLA [46]. For clinical specimens, sections (4 µm) were cut from formalin-fixed paraffin-embedded tissue.

PLA was performed after antigen retrieval (only in tissue samples; heat-induced epitope retrieval, 0.01 M citrate buffer pH 6.0). For cells and tissues, after protein blocking (Olink Bioscience, Sweden), permeabilization and overnight incubation with primary antibodies (goat anti-human VEGFR-3 and rabbit anti-PI3K p85), PLA was performed as per the manufacturer’s protocol [36] using the PLA PLUS and MINUS probes for goat and rabbit and the Duolink detection kit 613 (Olink Bioscience). DAPI was included to stain nuclei and anti-CD31 antibody was used to identify endothelial cells. For tissue sections, anti-CD31 antibody was replaced with anti-podoplanin antibody to identify lymphatic vessels. Duolink mounting medium (Olink Bioscience) was used to coverslip cell/tissue sections and staining evaluated using a Nikon C1 confocal microscope (Japan). Images show maximum intensity Z projection. The number of red PLA spots/nucleus was quantified using Olink Image Analysis software (Centre for Image Analysis, Uppsala University, Sweden).

Refer to Materials S1 for supplemental methods.

### Statistical Analyses

Data obtained in the microchemotaxis assays and Western blots were analyzed using one-way analysis of variance (ANOVA) followed by Bonferroni post test for multiple comparisons. The tube formation assays were analyses using two-way ANOVA. Data obtained from the *in situ* PLA was analyzed using t-test. The results are presented as mean ± s.e.m., except for PLA data from clinical specimens where the mean number of PLA signals per lymphatic vessel is shown for each sample and the group median indicated. *P*<0.05 was considered statistically significant. All calculations were performed using GraphPad Prism 5.0 (GraphPad Software, CA).

## Supporting Information

Figure S1 **IMC1121b inhibits VEGFR-2 phosphorylation induced by VEGF-A in LECs.** Western blotting analysis of total and phosphorylated VEGFR-2 (∼190 kDa) in LECs following 15 minute treatment with VEGF-A (25 ng/ml) and 1 hour treatment withIMC-1121b and hF4-3C5. Total and phospho-VEGFR-2 antibodies were used at 1 µg/ml. Control serum-free vehicle (IgG) treated LEC lysate is indicated by ‘0’.
(TIF)

Figure S2 **VEGF ligands do not induce phosphorylation of mTOR, whereas VEGF-C induces PI3K and PKC-dependent Erk1/2 phosphorylation via VEGFR-3 in LECs**. *A*) VEGF ligands: VEGF-A (100 ng/ml), VEGF-C (100 ng/ml), VEGF-C156S (250 ng/ml), VEGF-D (250 ng/ml) and VEGF-E (100 ng/ml) had no stimulatory effect on pS2448 mTOR protein expression. HEK293 cell lysate was used as positive control, indicated as ‘293’. *B*, Western blotting analysis of phosphorylated Erk1/2 in LECs following ligand stimulation: HGF (400 ng/ml, VEGF-A (100 ng/ml), VEGF-C (100 ng/ml), VEGF-C156S (250 ng/ml), VEGF-D (250 ng/ml) and VEGF-E (100 ng/ml); Time course for Erk1/2 phosphorylation in LECs after VEGF-C (100 ng/ml) and VEGF-A (100 ng/ml) treatment. *C*, Concentration-dependent phosphorylation of Erk1/2 in LECs after stimulation with VEGF-C for 15 minutes. *D*, Effect of inhibition of PI3K (AS252424), Raf/MEK (PD98059), or PKC (GF109203X) on Erk1/2 phosphorylation in LECs in response to VEGF-C. *E*, Phosphorylation of Erk1/2 in LECs following stimulation with VEGF-A or VEGF-E for 15 minutes at indicated concentrations. *F*, Phosphorylation of Erk1/2 in LECs following stimulation with VEGF-A or VEGF-E at indicated times. *G*, Effects of inhibition of VEGFR-3 (hF4-3C5), VEGFR-2 (IMC-1121b) or VEGFR-1 (IMC-18F1) on LEC Erk1/2 phosphorylation in response to VEGF-C. Serum-free vehicle (IgG) treated LEC lysate is indicated by ‘0’ in all blots.
(TIF)

Figure S3 **Effect of PLCγ1 knock-down on Akt (S473) phosphorylation in LECs.** Silencing PLCγ1 in LECs using siRNA had no effect on Akt (S473) phosphorylation in response to VEGF-C (100 ng/ml). Non-targeting control (NTC) was used as a negative control. Control untreated LEC whole cell lysate is indicated by (WCL). Vehicle-treated LEC is indicated by ‘−’ and VEGF-C treated is indicated by ‘+’. Densitometry analysis is shown in italics; where integrated intensity of siRNA PLCγ1 lysates was compared to that of the NTC each sample, and then expressed as fold change in integrated density.
(TIF)

Figure S4 **Characterization of isolated LEC by immunocytochemistry.** Immunocytochemistry staining shows VEGFR-3 (*left panel*, *green*), LYVE-1 (*middle panel, green*) and PROX-1 (*right panel, green*) staining in human LECs; DAPI staining is shown in blue. Bar, 50 µm.
(TIF)

Table S1 **Lymph node and clinicopathological status of small cell lung carcinoma patient cohort.**
(DOCX)

Materials S1 **Supplemental Methods.**
(DOC)
