# Supplementary figures and images for: Vascular Endothelial Growth Factor Receptor-3 Directly Interacts with Phosphatidylinositol 3-Kinase to Regulate Lymphangiogenesis
Source: PLoS One. 2012 Jun 22;7(6):e39558. doi: 10.1371/journal.pone.0039558 (PMC3382126; doi:10.1371/journal.pone.0039558)

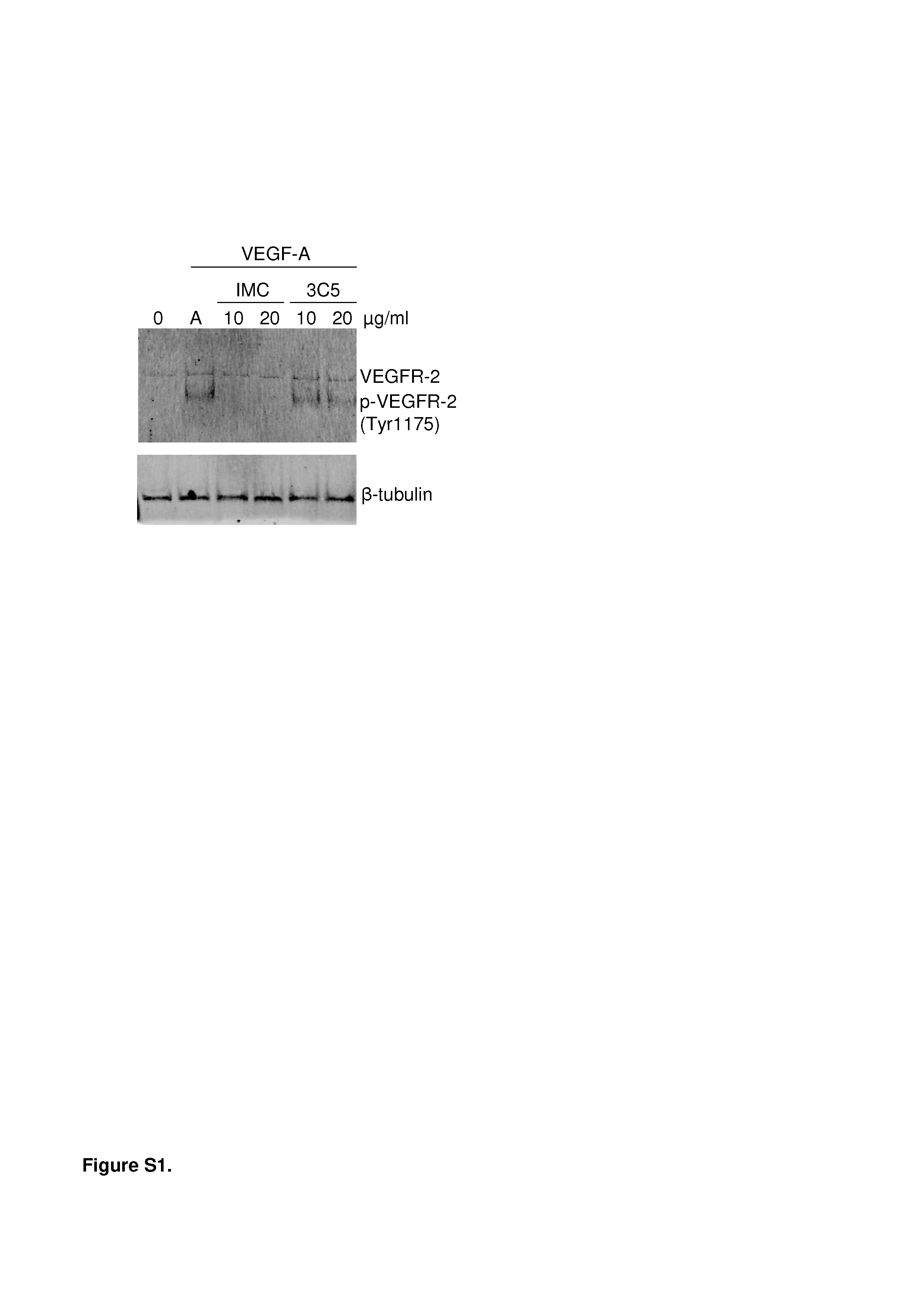

Supplement: Figure S1 — IMC1121b inhibits VEGFR-2 phosphorylation induced by VEGF-A in LECs. Western blotting analysis of total and phosphorylated VEGFR-2 (∼190 kDa) in LECs following 15 minute treatment with VEGF-A (25 ng/ml) and 1 hour treatment withIMC-1121b and hF4-3C5. Total and phospho-VEGFR-2 antibodies were used at 1 µg/ml. Control serum-free vehicle (IgG) treated LEC lysate is indicated by ‘0’. (TIF) [file pone.0039558.s001.tif]

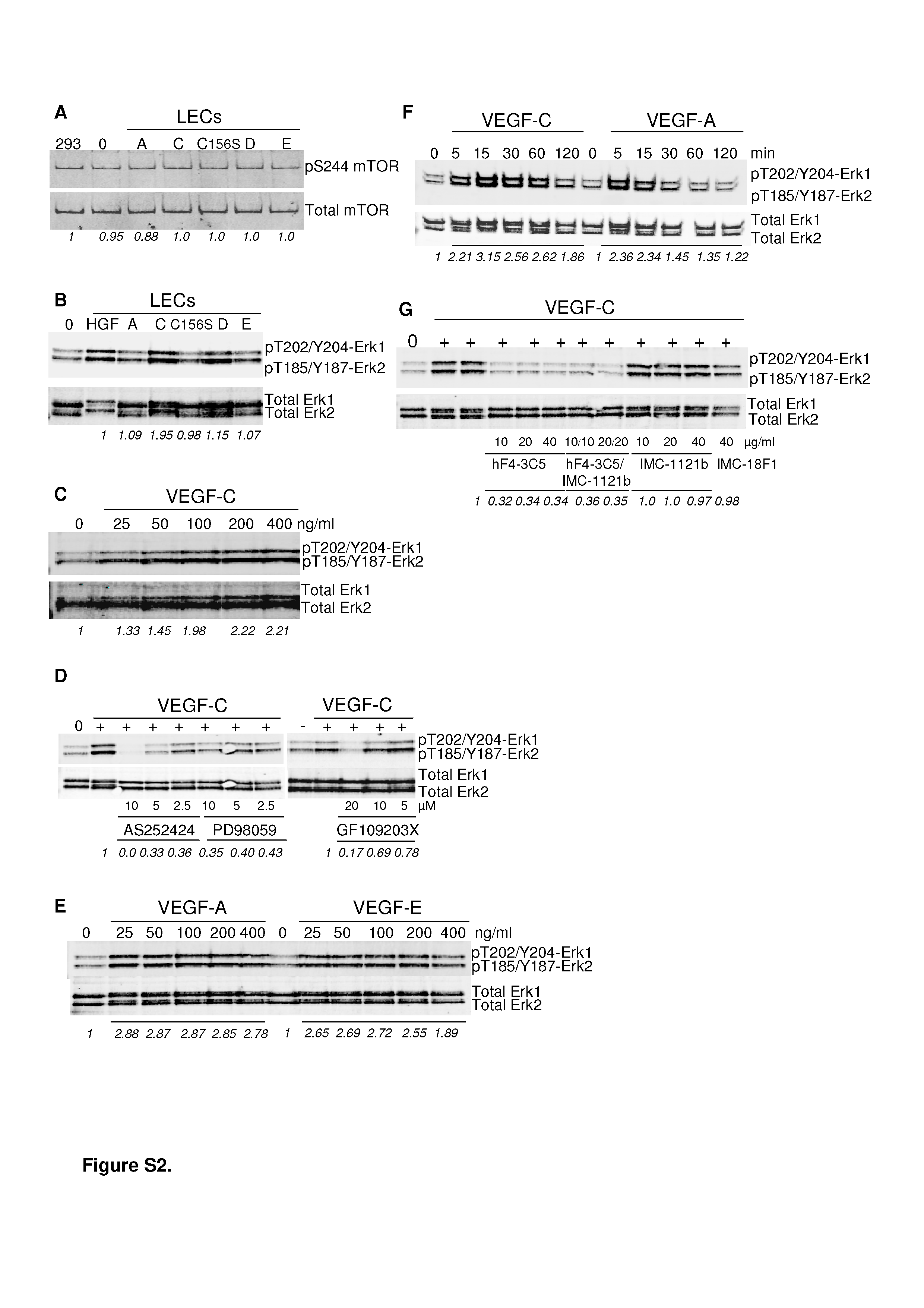

Supplement: Figure S2 — VEGF ligands do not induce phosphorylation of mTOR, whereas VEGF-C induces PI3K and PKC-dependent Erk1/2 phosphorylation via VEGFR-3 in LECs. A) VEGF ligands: VEGF-A (100 ng/ml), VEGF-C (100 ng/ml), VEGF-C156S (250 ng/ml), VEGF-D (250 ng/ml) and VEGF-E (100 ng/ml) had no stimulatory effect on pS2448 mTOR protein expression. HEK293 cell lysate was used as positive control, indicated as ‘293’. B, Western blotting analysis of phosphorylated Erk1/2 in LECs following ligand stimulation: HGF (400 ng/ml, VEGF-A (100 ng/ml), VEGF-C (100 ng/ml), VEGF-C156S (250 ng/ml), VEGF-D (250 ng/ml) and VEGF-E (100 ng/ml); Time course for Erk1/2 phosphorylation in LECs after VEGF-C (100 ng/ml) and VEGF-A (100 ng/ml) treatment. C, Concentration-dependent phosphorylation of Erk1/2 in LECs after stimulation with VEGF-C for 15 minutes. D, Effect of inhibition of PI3K (AS252424), Raf/MEK (PD98059), or PKC (GF109203X) on Erk1/2 phosphorylation in LECs in response to VEGF-C. E, Phosphorylation of Erk1/2 in LECs following stimulation with VEGF-A or VEGF-E for 15 minutes at indicated concentrations. F, Phosphorylation of Erk1/2 in LECs following stimulation with VEGF-A or VEGF-E at indicated times. G, Effects of inhibition of VEGFR-3 (hF4-3C5), VEGFR-2 (IMC-1121b) or VEGFR-1 (IMC-18F1) on LEC Erk1/2 phosphorylation in response to VEGF-C. Serum-free vehicle (IgG) treated LEC lysate is indicated by ‘0’ in all blots. (TIF) [file pone.0039558.s002.tif]

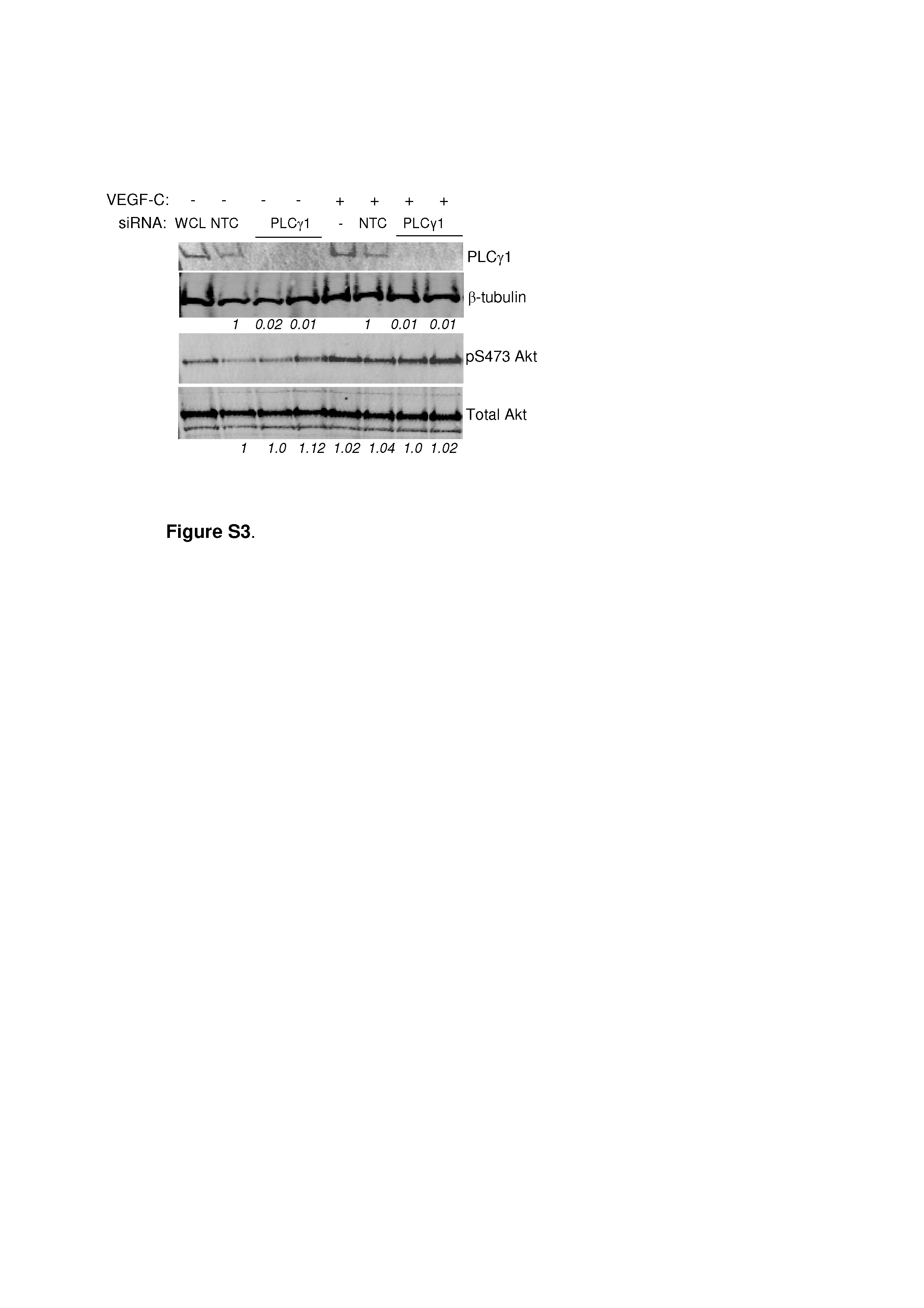

Supplement: Figure S3 — Effect of PLCγ1 knock-down on Akt (S473) phosphorylation in LECs. Silencing PLCγ1 in LECs using siRNA had no effect on Akt (S473) phosphorylation in response to VEGF-C (100 ng/ml). Non-targeting control (NTC) was used as a negative control. Control untreated LEC whole cell lysate is indicated by (WCL). Vehicle-treated LEC is indicated by ‘−’ and VEGF-C treated is indicated by ‘+’. Densitometry analysis is shown in italics; where integrated intensity of siRNA PLCγ1 lysates was compared to that of the NTC each sample, and then expressed as fold change in integrated density. (TIF) [file pone.0039558.s003.tif]

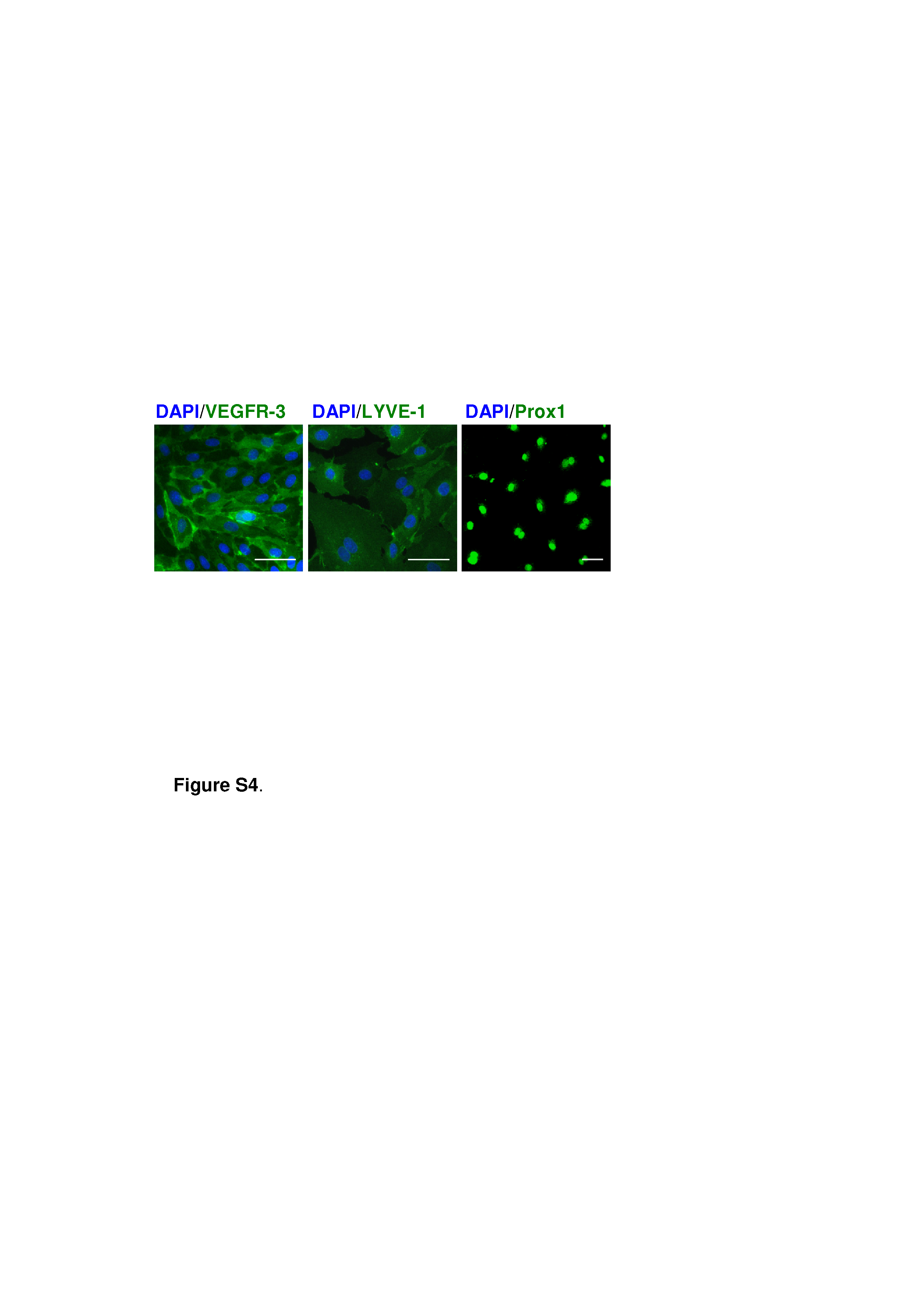

Supplement: Figure S4 — Characterization of isolated LEC by immunocytochemistry. Immunocytochemistry staining shows VEGFR-3 (left panel, green), LYVE-1 (middle panel, green) and PROX-1 (right panel, green) staining in human LECs; DAPI staining is shown in blue. Bar, 50 µm. (TIF) [file pone.0039558.s004.tif]
