# Supplementary material for: Vascular Endothelial Growth Factor Receptor-3 Directly Interacts with Phosphatidylinositol 3-Kinase to Regulate Lymphangiogenesis
Source: PLoS One. 2012 Jun 22;7(6):e39558. doi: 10.1371/journal.pone.0039558 (PMC3382126; doi:10.1371/journal.pone.0039558)
Supplement: Table S1 — Lymph node and clinicopathological status of small cell lung carcinoma patient cohort. (DOCX) [file pone.0039558.s005.docx]

**Table S1** Lymph node and clinicopathological status of small cell lung carcinoma patient cohort

| **Variable** | **LN-** | **LN+** | ***P* value** |
| --- | --- | --- | --- |
| **% male** | 89 | 90 | 1 |
| **Mean age at surgery** (years) | 69  (range 54-82) | 68  (range 52-79) | 0.8312 |
| **Mean primary tumor diameter** (mm) | 37  (range 10-96) | 49.6  (range 4-100) | 0.3917 |

LN = lymph node
