# Supplementary material for: Vascular Endothelial Growth Factor Receptor-3 Directly Interacts with Phosphatidylinositol 3-Kinase to Regulate Lymphangiogenesis
Source: PLoS One. 2012 Jun 22;7(6):e39558. doi: 10.1371/journal.pone.0039558 (PMC3382126; doi:10.1371/journal.pone.0039558)
Supplement: Materials S1 — Supplemental Methods. (DOC) [file pone.0039558.s006.doc]

**SUPPLEMENTAL MATERIAL**

**Supplemental Methods**

#### LEC treatment, immunoprecipitation, and Western blotting analyses were performed as described in the main manuscript.

Phosphorylated VEGFR-2 (Tyr1175, Clone: 19A10, Cat. No. 2478S) antibody was obtained from Cell Signaling Technology (MA).

#### Knockdown of PLCγ1 in LECs using siRNA and PLA assays were performed as described in the main manuscript.
